# Chemical cues that attract cannibalistic cane toad (*Rhinella marina*) larvae to vulnerable embryos

**DOI:** 10.1038/s41598-021-90233-3

**Published:** 2021-06-15

**Authors:** Michael R. Crossland, Angela A. Salim, Robert J. Capon, Richard Shine

**Affiliations:** 1 School of Life and Environmental Sciences A08, University of Sydney, Sydney, NSW 2006 Australia; 2 Division of Chemistry and Structural Biology, Institute for Molecular Bioscience, The University of Queensland, St Lucia, QLD 4072 Australia; 3 Department of Biological Sciences, Macquarie University, Sydney, NSW 2109 Australia

**Keywords:** Ecology, Zoology

## Abstract

Chemical cues produced by late-stage embryos of the cane toad (*Rhinella marina*) attract older conspecific larvae, which are highly cannibalistic and can consume an entire clutch. To clarify the molecular basis of this attraction response, we presented captive tadpoles with components present in toad eggs. As previously reported, attractivity arises from the distinctive toxins (bufadienolides) produced by cane toads, with some toxins (e.g., bufagenins) much stronger attractants than others (e.g., bufotoxins). Extracts of frozen toad parotoid glands (rich in bufagenins) were more attractive than were fresh MeOH extracts of the parotoid secretion (rich in bufotoxins), and purified marinobufagin was more effective than marinobufotoxin. Cardenolide aglycones (e.g., digitoxigenin) were active attractors, whereas C-3 glycosides (e.g., digoxin, oubain) were far less effective. A structure–activity relationship study revealed that tadpole attractant potency strongly correlated with Na^+^/K^+^ ATPase inhibitory activity, suggesting that tadpoles monitor and rapidly react to perturbations to Na^+^/K^+^ ATPase activity.

## Introduction

For many types of organisms, conspecifics are among the most important predators^1,2^. Cannibalism typically involves older (larger) individuals consuming younger (smaller) members of their own species, and can be frequent enough to massively impact overall abundances. For example, field trials in tropical Australia showed that larval cane toads (*Rhinella marina*) consumed more than 99% of eggs that were laid^3,4^. That mortality is important from a wildlife-management perspective, because cane toads are an invasive taxon in Australia, and have imperilled several species of native predators through lethal toxic ingestion^5^. The high frequency of cannibalism means that this interaction has strong consequences for population densities of toads and also, provides a potential tool by which managers might be able to reduce toad abundance. By luring toad larvae into funnel traps baited with attractant chemicals, we may be able to eradicate this invasive pest from waterbodies^6,7^.

In previous work, we have shown that the chemical basis for attraction of toad larvae to developing eggs involves the distinctive toxins (bufadienolides) produced by this species^6^. As a result, secretions from the adult toad’s parotoid glands (the main site of storage of these defensive toxins) can be used as “bait” to attract toad larvae^6,7^. The present study extends our work on this system to further clarify the chemical basis of the attractant cue and the mechanisms by which it operates. To achieve this aim we conducted laboratory trials in which we exposed captive larvae to a range of concentrations of several chemicals that were judged likely to elicit the attractant response.

We also exposed the larvae to extracts obtained from different life stages of cane toads (eggs, early-development and late-development tadpoles), from frozen parotoid glands and from fresh parotoid secretion, to compare attractant responses to the types of compounds present in each extract.

## Materials and methods

### Parotoid gland extract preparation

**Figure 1:**
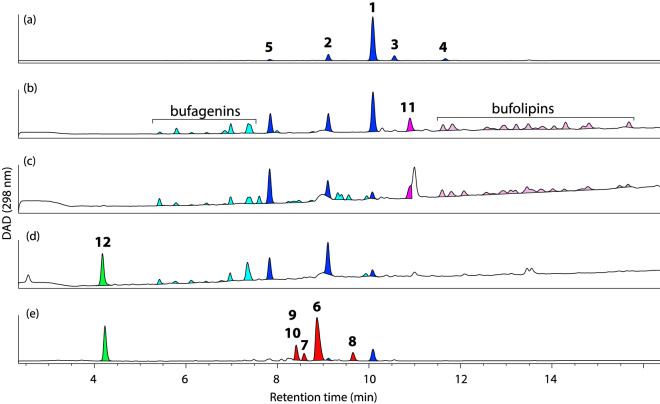
Analytical HPLC (298 nm) chromatogram of extracts obtained from (**a**) frozen parotoid gland, (**b**) eggs, (**c**) early-development tadpole, (**d**) late-development tadpole and (**e**) fresh parotoid secretion of cane toads, *Rhinella marina* [HPLC condition: Agilent Zorbax C_8_ column, 5 μm, 4.6 × 150 mm, 1 mL/min flow rate 15 min gradient elution from 90% H_2_O in MeCN, to 100% MeCN, with a constant 0.01% TFA in MeCN modifier]. Highlights: light blue = unspecified bufagenins (MW 400–432); light pink = unspecified bufolipins (MW 630–700); blue = bufagenins **1–5**; red = bufotoxins **6–10**; pink = bufolipin **11**. Structures for **1–11** are shown in Fig. 2, and were assigned on the basis of spectroscopic analysis and comparisons with authentic standards.

Adult cane toads (obtained in south-eastern Queensland, December 2018) were killed humanely using the cool/freeze method^8^ and stored at − 20 °C. Parotoid glands (54 g) excised from 23 thawed toads were macerated in H_2_O (250 mL) with a commercial blender, and filtered through a bed of Celite 545. The filtrate was concentrated in vacuo at 40 °C, and was partitioned into ethyl acetate (EtOAc) and H_2_O solubles. The EtOAc extract (750 mg) containing mostly bufagenins (Fig. 1a) was used in the attractant assay without further purification.

### Egg extract preparation

Cane toad eggs obtained from two laboratory-laid clutches (see method below, Northern Territory, October 2010) were stored at − 20 °C until extraction. Frozen eggs were freeze-dried to yield dry material (1.5 g) that was extracted overnight at room temperature with 90:10 MeOH:H_2_O (100 mL). The resulting solvent extract was concentrated in vacuo at 40 °C to give a crude material (251 mg) which was partitioned into EtOAc and H_2_O solubles. The EtOAc (160 mg) extract containing mostly bufagenins and bufolipins (Fig. 1b) was used in the attractant assay without further purification.

### Early-development tadpole extract preparation

Early developmental stage cane toad tadpoles were collected live from the wild (Northern Territory, March 2010), and stored at − 18 °C until extraction. Frozen tadpoles were freeze-dried to yield dry material (2.6 g) that was extracted overnight at room temperature with 90:10 MeOH:H_2_O (100 mL). The resulting solvent extract was concentrated in vacuo at 40 °C to give a crude material (1062 mg), which was partitioned into n-BuOH and H_2_O solubles. The BuOH extract (582 mg) containing mostly bufagenins and bufolipins (Fig. 1c) was used in the attractant assay without further purification.

### Late-development tadpole extract preparation

Mid to late developmental stage tadpoles were collected live from the wild (Northern Territory, December 2010), and stored at − 18 °C until extraction. Frozen tadpoles were freeze-dried to give dry material (13.4 g) that was extracted overnight at room temperature with 90:10 MeOH:H_2_O (100 mL). The resulting solvent extract was concentrated in vacuo at 40 °C to give a crude material (6899 mg), which was partitioned into n-BuOH and H_2_O solubles. The BuOH extract (3885 mg) containing mostly bufagenins (Fig. 1d) was used in the attractant assay without further purification.

### Parotoid secretion extract preparation

Parotoid secretion was obtained from a live adult toad (Northern Territory, August 2011) by mechanical compression of the parotoid gland directly into MeOH, which following concentration in vacuo yielded a crude MeOH extract (26.2 mg). The crude MeOH extract containing mostly bufotoxins (Fig. 1e) was used in the attractant assay without further purification.

### Pure compounds preparation

**Figure 2:**
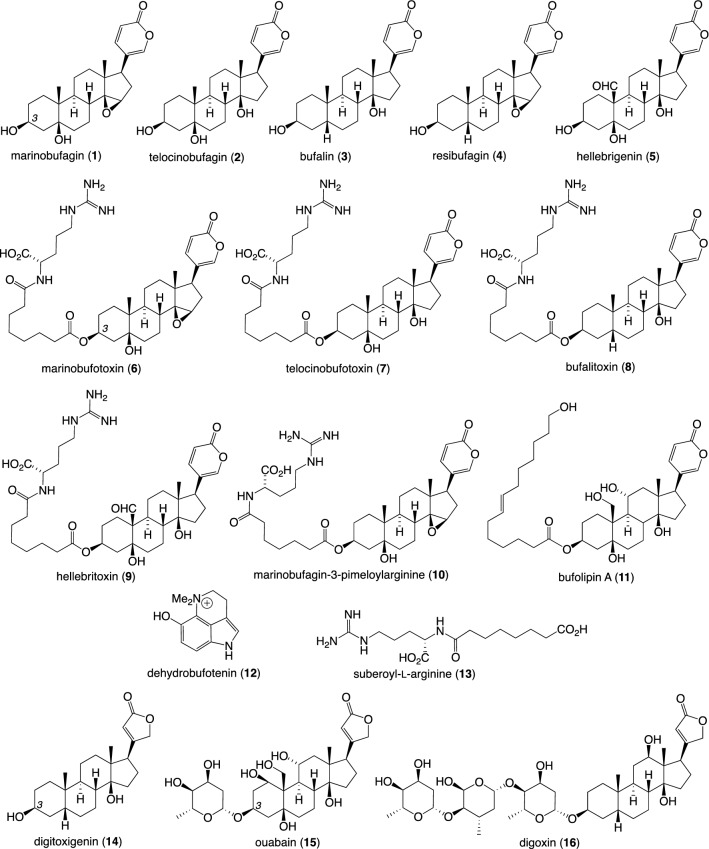
Compounds identified in different stages of cane toad (*Rhinella marina*) (**1**–**13**) and plant derived cardenolides (**14**–**16**).

Marinobufagin (**1**), marinobufotoxin (**6**) and suberoyl-l-arginine (**13**) were obtained from our in-house pure compound library, and their purities were confirmed by LCMS, HRMS and NMR (see Supporting Information for ^1^H NMR spectra of the pure compounds). Plant cardenolides: digitoxigenin (**14**), ouabain (**15**) and digoxin (**16**) (Fig. 2) were purchased from Sigma Aldrich and were used in the attractant assay without further purification.

### Chemical analyses

Analytical HPLC was performed using an Agilent 1100 series module equipped with a diode array detector on an Agilent Zorbax Stable Bond C_8_ column (4.6 × 150 mm, 5 μm), 1 mL/min flow rate, 15 min gradient elution from 90% H_2_O in MeCN to 100% MeCN with a constant 0.01% TFA in MeCN modifier. All analytes were prepared in MeOH stock solutions (1 mg/mL) and an aliquot (10 μL) used for each analysis. HPLC chromatograms were monitored at 298 nm (the α-pyrone chromophore common to all bufadienolides). Compounds **1**–**12** (Fig. 2) present in the extracts were identified by LC-DAD-ESIMS and comparison with authentic standards (see Supporting Information Table S1). LC-DAD-ESIMS (Liquid Chromatography coupled to Diode Array Detector and Electrospray Ionization Mass Spectra) was acquired using an Agilent 1100 Series LC/MSD mass detector in both positive and negative modes using Agilent Zorbax Stable Bond C_8_ column (4.6 × 150 mm, 5 μm) with 1 mL/min flow rate, 15 min gradient elution from 90% H_2_O in MeCN to 100% MeCN with a constant 0.05% formic acid in MeCN modifier.

### Bait preparations

Stock solutions of all attractant extracts were prepared in MeOH (20, 2.0 and 0.20 mg/mL concentrations), with a fixed volume (0.5 mL) of each loaded onto porous ceramic rings (Majestic Aquariums, Sydney, NSW) to give a series of loadings per ceramic ring (10, 1.0 and 0.1 mg) per attractant extract preparation. Stock solutions were also prepared for all pure compound attractants in MeOH (5.0 and 0.5 mM) with a fixed volume (0.5 mL) of each loaded onto porous ceramic rings to give a series of loadings per ceramic ring (2.5 and 0.25 µmoles) per attractant pure compound preparation (marinobufagin, 1.00 and 0.10 mg; digitoxigenin, 0.94 and 0.094 mg; marinobufotoxin, 1.78 and 0.178 mg; ouabain octahydrate, 1.82 and 0.182 mg; digoxin, 1.95 and 0.195 mg; suberoyl-l-arginine, 0.825 and 0.0825 mg per ceramic ring, respectively). Negative controls were ceramic rings loaded with MeOH (0.5 mL/ring) only. All impregnated ceramic rings were left in the fume-hood overnight at room temperature to allow the MeOH to evaporate, and to fix the attractants to the ceramic matrix.

### Toad breeding

Adult toads were collected from the Adelaide River floodplain, near the city of Darwin in tropical Australia, and the animals were held in outdoor enclosures at The University of Sydney Tropical Ecology Research Facility at Middle Point, Northern Territory (12°34.73′S, 131°18.85″E). Breeding was induced by injection of the synthetic gonadotrophin leuprorelin acetate (Lucrin, Abbott Australasia). Females were injected with 0.75 mL doses of 0.25 mg/mL, while males were injected with doses of 0.25 mL^4,9^. Toads were injected just prior to sunset, and the pairs were placed in 70 L plastic tubs set on an angle with 8 L water. The following morning, eggs were collected and placed in 18 L tanks holding 9 L aerated water. When eggs developed into free-swimming tadpoles (Gosner^10^ stage 25), tadpoles were transferred to outdoor 750 L mesocosms located in a shaded area. Tadpoles were fed algae wafers (Kyorin, Japan) ad libitum daily, with 50% of the water in mesocosms changed every 3 days. Tadpoles (stage 30–39) were haphazardly selected from mesocosms for use in attraction trials as required.

### Attraction trials

Attraction trials were conducted in a covered outdoor enclosure exposed to ambient temperature between 0930 and 1700 hours (maximum daily water temperature range over all trials: 26–32 °C). Each trial used plastic pools (1 m diameter) filled with 90 L of well water. Within each pool we placed two plastic traps (175 mm × 120 mm × 70 mm), each of which had a funnel (1 cm diameter) attached to one side. The traps were positioned in the centre of the pool 5 cm apart, with the funnels facing outward. Each pool was stocked with 50 tadpoles from a single clutch. Tadpoles were allowed to settle for 2 h, after which we randomly allocated treatments to traps (i.e., control or chemical). A single bait was added to each trap, and the number of tadpoles within each trap was counted hourly for 6 h. Water temperature was measured at hourly intervals using a hand-held thermometer.

Attraction responses to 26 combinations of chemical/concentration were tested, using a total of nine tadpole clutches. Each concentration of each chemical was tested using 4–7 tadpole clutches. The tadpole clutches used for each trial were chosen randomly, with the proviso that they had not been previously tested with the same chemical concentration. Individual tadpoles and baits were used only once in trials.

### Statistical analysis

We analysed tadpole attraction as a binomial response (trap preference: chemical trap vs control trap) using logistic regression^11^ in R^12^, package MASS:glmmPQL). Models were based on the quasibinomial distribution to account for overdispersion of data, with Treatment (control vs. chemical) and Time (hourly intervals) as fixed effects. Random effects were accounted for by nesting trap within pool and responding tadpole clutch. We did not apply Bonferroni corrections to treatment p values due to the highly subjective nature of deciding when to apply such corrections^13,14^, see both papers for further problems with use of Bonferroni corrections). Rather, we provide unadjusted treatment p values in association with effect sizes (i.e., odds ratio of trap preference; this being a more meaningful indicator of biological significance) to interpret our attraction results^13,14^.

### Ethics approval

This research was approved under permit 6033 from the University of Sydney Animal Care Committee. All methods were performed in accordance with the relevant guidelines and regulations, including ARRIVE guidelines.

### Consent for publication

All authors agree to publication of this work.

## Results

Chemical analysis showed that the extract of parotoid glands contained only bufagenins **1**–**5**, with marinobufagin (**1**) present at ~ 80% w/w (Fig. 1a). Extracts of eggs and early tadpole showed very similar chemical contents, with the only significant difference being the higher level of marinobufagin present in the egg extracts (Fig. 1b,c). These two extracts also contained bufolipin A (**11**) and other minor bufolipins (bufadienolide fatty acid esters), which are characterised by their late eluting times in reversed-phase HPLC and high MW (*m/z* 600–700). The extract of late tadpoles was dominated by bufagenins (with telocinobufagin (**2**) and hellebrigenin (**5**) as major components) and dehydrobufotenin (**12**) (Fig. 1d). The extract of fresh parotoid secretion showed a very different chemical profile to those obtained from eggs, tadpoles and frozen parotoid glands, with marinobufotoxin (**6**) as the major component and a mixture of bufotoxins **7**–**10** and marinobufagin (**1**) as minor components (Fig. 1e).

**Figure 3:**
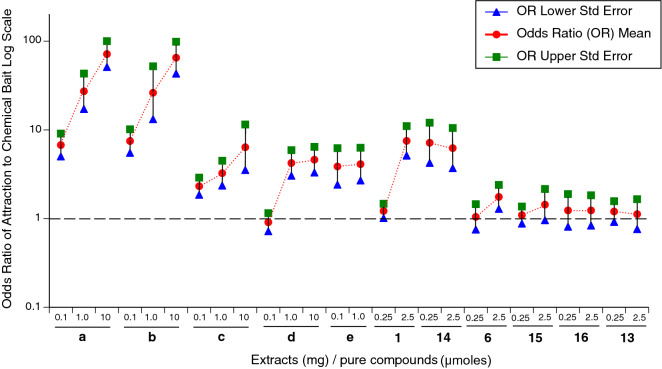
The degree to which cane toad (*Rhinella marina*) tadpoles were attracted to a range of stimuli from conspecifics: (**a**) frozen parotoid gland, (**b**) toad eggs, (**c**) early-development tadpole extracts, (**d**) late-development tadpole extracts, (**e**) fresh parotoid secretion, and pure compounds: (**1**) marinobufagin, (**14**) digitoxigenin, (**6**) marinobufotoxin, (**15**) ouabain, (**16**) digoxin, (**13**) suberoyl-l-arginine, as quantified by the numbers captured in baited versus control traps. Data plotted are the odds of a tadpole selecting a trap baited with chemical compared to a control bait. The dashed horizontal line at odds ratio of attraction at 1 shows the null expectation (no disparity between baited and control traps) if there is no significant attraction. The points show means and standard errors. Treatments with the same chemical composition, differing only in concentration, are linked by dotted lines.

For all trapping sessions, the numbers of tadpoles inside traps increased through time (p < 0.0001 in all instances). Treatments (chemical × concentration) differed in terms of attractiveness, with statistically significant attractiveness documented for one or more concentrations of the following chemical baits: extract of frozen parotoid glands, extract of eggs, extract of early-development tadpoles, extract of late-development tadpoles, extract of fresh parotoid secretion, marinobufagin (**1**) and digitoxigenin (**14**). In contrast, no significant attraction was detected for marinobufotoxin (**6**), ouabain (**15**), digoxin (**16**) or suberoyl-l-arginine (**13**) (Table 1; Fig. 3).

**Table 1:** Logistic regression results for attraction (trap preference: chemical vs control) by toad tadpoles. Model structure had main effects of Treatment and Time, with trap nested within pool and responding tadpole clutch as random effects. The main effect of Time was p < 0.0001 for all chemicals. *p < 0.05 for Treatment effect.

Chemical bait	Treatment		
	t	df	p
Parotoid gland frozen 0.1 mg	6.422	3	0.0077*
Parotoid gland frozen 1 mg	7.168	4	0.0020*
Parotoid gland frozen 10 mg	12.702	4	0.0002*
Egg extract 0.1 mg	6.558	3	0.0072*
Egg extract 1 mg	4.751	5	0.0051*
Egg extract 10 mg	10.082	5	0.0002*
Early tadpole extract 0.1 mg	3.755	4	0.0199*
Early tadpole extract 1 mg	3.651	5	0.0147*
Early tadpole extract 10 mg	3.131	5	0.0259*
Late tadpole extract 0.1 mg	− 0.404	4	0.7071
Late tadpole extract 1 mg	4.349	4	0.0122*
Late tadpole extract 10 mg	4.565	4	0.0103*
Parotoid secretion MeOH 0.1 mg	2.853	4	0.0462*
Parotoid secretion MeOH 1 mg	3.320	4	0.0294*
Marinobufagin 0.25 µmoles	1.057	5	0.3388
Marinobufagin 2.5 µmoles	5.268	6	0.0019*
Digitoxigenin 0.25 µmoles	3.768	5	0.0130*
Digitoxigenin 2.5 µmoles	3.500	4	0.0249*
Marinobufotoxin 0.25 µmoles	0.136	5	0.8975
Marinobufotoxin 2.5 µmoles	1.801	4	0.1461
Ouabain 0.25 µmoles	0.410	6	0.6962
Ouabain 2.5 µmoles	0.894	6	0.4058
Digoxin 0.25 µmoles	0.501	5	0.6377
Digoxin 2.5 µmoles	0.539	5	0.6132
Suberoyl-l-arginine 2.5 µmoles	0.681	5	0.5262
Suberoyl-l-arginine 2.5 µmoles	0.296	4	0.7816

Extracts of frozen parotoid glands and eggs exhibited the highest levels of attraction (the odds of a tadpole choosing a trap when it was baited with these chemicals were 7 to 72 times those if it was baited with a control bait), whereas extracts from early-stage tadpoles, late-stage tadpoles and fresh parotoid secretions were less attractive (a tadpole was ≤ 6 times more likely to choose a trap baited with these chemicals than a control trap) (Fig. 3). Pure compounds** 1** and **14** elicited moderate attraction scores (a tadpole was up to 8 times more likely to choose a chemical-baited trap than a control trap), but compounds **6**, **13**, **15**–**16** did not significantly attract tadpoles (odds ratio estimates ~ 1, indicating a tadpole was just as likely to choose a chemical-baited trap as a control trap; Fig. 3).

## Discussion

The current study supports and extends previous research on the attraction response of toad tadpoles towards bufadienolides (bufagenins and bufotoxins) present in the eggs and parotoid secretions of this species. In previous studies, extracts were obtained by allowing eggs to develop in water, and the entire composite solution was then added directly to the tank to measure attraction responses in the laboratory^15^, or in the case of parotoid secretion, was freshly obtained from live adult cane toads and the exudate was placed directly in funnel traps for field trials^5^. In the current study, all extracts were applied on solid matrices (porous ceramic rings), which were then placed in funnel traps to allow for the slow release of bufadienolides into the water. In this study, we prepared extracts of parotoid secretions obtained from live adult cane toads, as well as extracts of parotoid glands dissected from frozen cane toads. In addition, we also tested the attraction response of toad tadpoles to extracts obtained from tadpoles in early and late development. Thus, we can correlate the degrees of attraction with the chemical composition of the extracts tested.

Our results (Fig. 3) showed that extracts from toad eggs and frozen parotoid glands elicited similar attraction responses in toad tadpoles when tested at three different concentrations. Of note, extracts from fresh parotoid secretion were less attractive than were those of frozen parotoid glands (odds of a tadpole choosing a chemical-baited trap over a control trap = 4 vs. 27, respectively, for 1 mg extract). This result prompted us to investigate the bufadienolide composition of these extracts to explain the difference in attraction responses. Our HPLC analysis (Fig. 1) showed that both frozen parotoid gland and egg extracts contain marinobufagin (**1**) as the major component. Fresh parotoid secretion extract showed a different bufadienolide profile, dominated by marinobufotoxin (**6**). We tested pure compounds **1** and **6** in attraction assays, and confirmed that **1** significantly attracted toad tadpoles while **6** was not active (Fig. 1). In combination, these results explain why the frozen parotoid gland was more attractive than the fresh parotoid secretion.

We also showed that ceramic baits containing extracts from frozen parotoid glands are good substitutes for the baits prepared from fresh parotoid secretion. Moreover, the ceramic bait has added advantages because it can be handled easily (as opposed to sticky parotoid secretion), is more difficult to be ingested accidentally (e.g., by children, domestic pets, or native fauna) and can be stored at room temperature for long periods of time.

Extracts from tadpoles, both early and late in development, elicited comparable responses at higher concentrations (1 and 10 mg), but the attraction response was less intense than was that elicited by extracts of eggs and frozen parotoid gland. This result might be due to the lower concentration of **1** in both extracts, or by the presence of “alarm chemicals” which induce avoidance in conspecific tadpoles^16^. Such alarm cues might send conflicting signals to tadpoles, thereby reducing the attraction response to bufadienolides. Toad tadpoles release specific alarm cues when injured, including suberic acid^16^. Although most of the suberic acid will partition into the water-soluble layer during extract preparation, trace amounts in the BuOH extract may be enough to elicit avoidance. Because suberic acid does not have a strong UV chromophore, its presence cannot be detected in the HPLC–DAD chromatogram (Fig. 1).

We also tested several plant-derived cardenolides (**14**–**16**), a group of cardioactive steroids featuring α-butenolide rather than the α-pyrone rings common to bufadienolides (Fig. 2). We found that cardenolide aglycones (e.g., digitoxigenin) elicited an attractant response, whereas the corresponding glycosides (e.g., ouabain and digoxin) did not. This result fits well with the fact that corresponding bufagenin “aglycones” (e.g., marinobufagin) elicited a far stronger attractant response than did C-3 acylated analogues (e.g., marinobufotoxin). These observations reveal that bufadienolides and cardenolides exhibit comparable attractant structure–activity relationships, and that this closely mirrors Na^+^/K^+^ ATPase inhibitory activity (i.e., the pharmacological basis of toad toxin toxicity in an ecological setting), suggesting that toad tadpoles monitor and rapidly react (are attracted) to perturbations in Na^+^/K^+^ ATPase activity. That is, tadpoles may recognise the presence of conspecific eggs based on the physiological response induced by those compounds rather than by the molecular structure of a natural chemical cue(s).

Lastly, we consider the implications of our results for management of invasive cane toads. We first observed the attraction behaviour of toad tadpoles to newly laid clutches of toad eggs^15^, suggesting that such eggs could be used as “bait” in traps to collect conspecific tadpoles. However, there are severe logistical challenges in finding enough toad eggs in the wild (or breeding them in a laboratory setting), precluding the widespread use of toad egg extracts as a toad tadpole attractant. Identifying the nature of the chemical cues involved (toad toxins) allowed us to simplify the trapping procedure by using toxins expressed from parotoid glands of adult toads^5^. The current study takes this result a step further, by showing that extracts sourced from frozen parotoid glands provide an even more effective attractant than do extracts from freshly-obtained parotoid glands. This is a practicable source of attractant cues, whereby parotoids can be excised from adult toads culled by community groups (e.g.^17^), and then frozen prior to solvent extraction. Ceramic baits infused with this extract can be stored at room temperature, and pose little risk of accidental ingestion. As evidence of its potential, over the last few years a community engagement and citizen science program (https://imb.uq.edu.au/canetoadchallenge) has successfully delivered this technology into the hands of the public, leading to the species-selective, environmentally sustainable and humane capture and eradication of many millions of tadpoles from local, managed waterways (dams, ponds, lakes, creeks and streams).

In conclusion, we have shown that extracts from frozen parotoid glands were very effective attractants of cane toad larvae due to the presence of bufadienolides (bufagenins). On the other hand, tadpole extracts, although rich in bufadienolides (bufagenins), were less effective as attractant baits, possibly because of the presence of alarm cues that induce avoidance in the cane toad larvae. Extracts from fresh parotoid secretion were less effective as attractant baits due to the presence of high amount of bufotoxins (C-3 acylated bufadienolides). The fact that digitoxigenin also elicited an attraction response in toad tadpoles suggest that plants rich in cardenolide aglycones (i.e., milkweed^18^) or bufadienolide aglycones (i.e., mother-of-millions^19^) may provide an alternative source of tadpole attractant.

## Supplementary Information

Supplementary Information.

## Data Availability

Data is available from Dryad Digital Repository 10.5061/dryad.3ffbg79hv.
