# Supplementary material for: Chemical cues that attract cannibalistic cane toad (Rhinella marina) larvae to vulnerable embryos
Source: Sci Rep. 2021 Jun 15;11:12527. doi: 10.1038/s41598-021-90233-3 (PMC8206116; doi:10.1038/s41598-021-90233-3)
Supplement: Supplementary file 1 — Supplementary Information. [file 41598_2021_90233_MOESM1_ESM.docx]

**Supporting Information**

**The Chemistry of Cannibalism: What Cues Attract Predatory Cane Toad (*Rhinella marina*) Larvae to Vulnerable Embryos?**

**Michael R. Crossland^1*^ ⋅ Angela A. Salim^2*^ ⋅ Robert J. Capon^2^ ⋅ Richard Shine^1,3^**

^1^School of Life and Environmental Sciences A08, University of Sydney, New South Wales 2006, Australia

^2^Division of Chemistry and Structural Biology, Institute for Molecular Bioscience, The University of Queensland, Queensland 4072, Australia

^3^Department of Biological Sciences, Macquarie University, New South Wales 2109, Australia

*Equal contribution as first authors

🖂 Richard Shine

[Rick.shine@mq.edu.au](mailto:Rick.shine@mq.edu.au)

**NMR spectra of pure compounds**


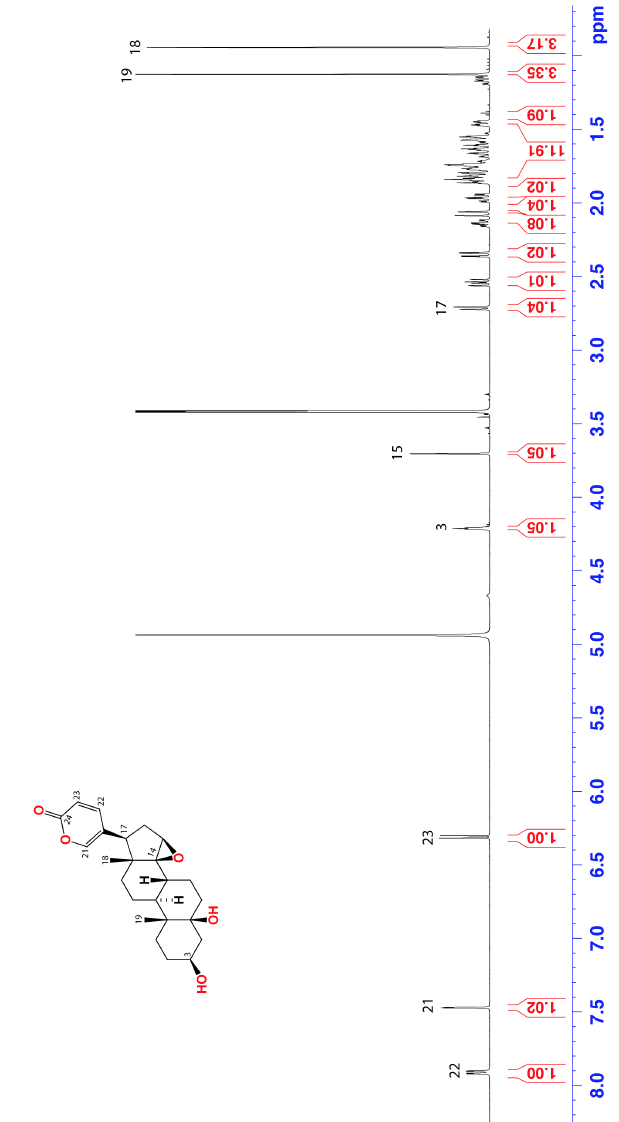


**Figure S1.** ^1^H NMR (CD_3_OD, 600 MHz) spectrum of marinobufagin (**1**)


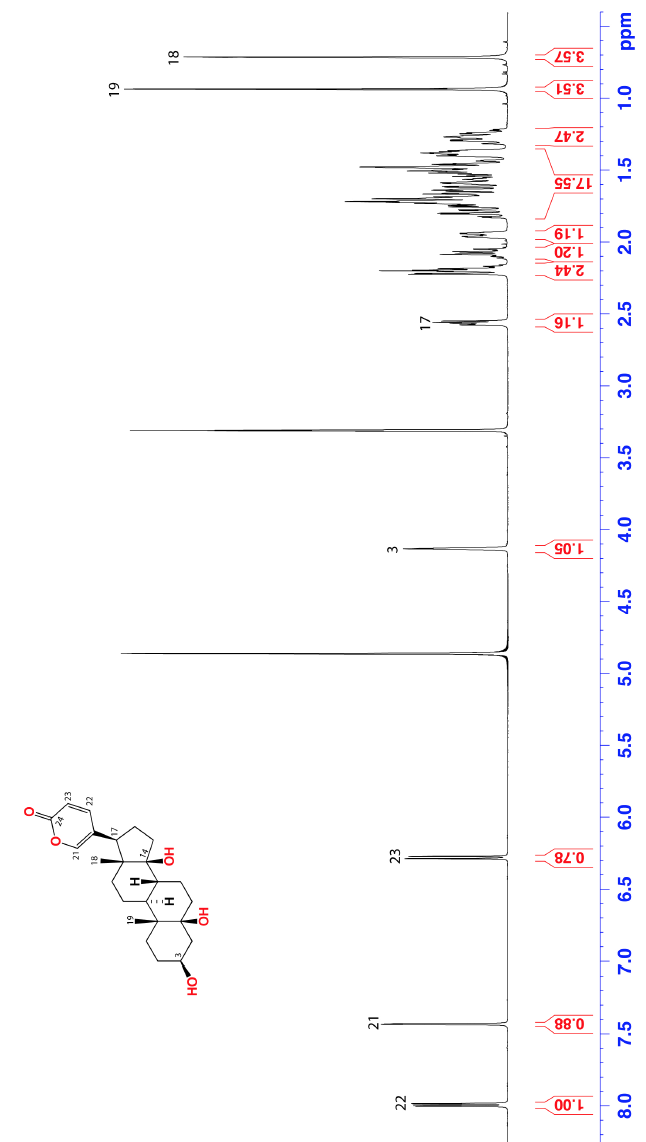


**Figure S2.** ^1^H NMR (CD_3_OD, 600 MHz) spectrum of telocinobufagin (**2**)


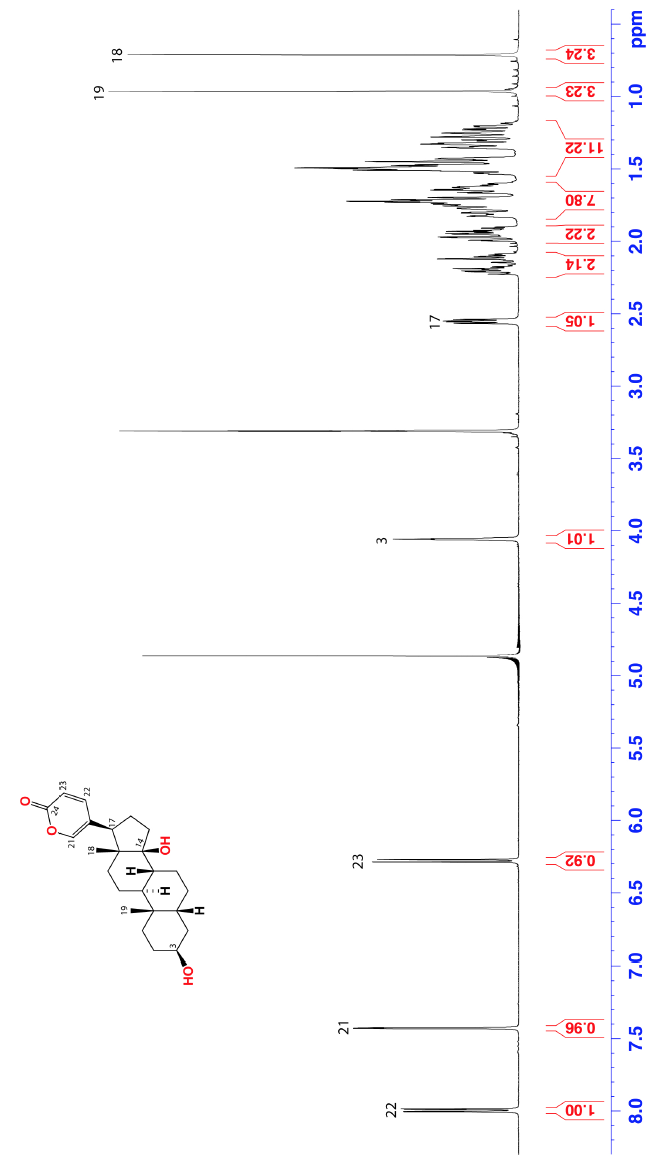


**Figure S3.** ^1^H NMR (CD_3_OD, 600 MHz) spectrum of bufalin (**3**)


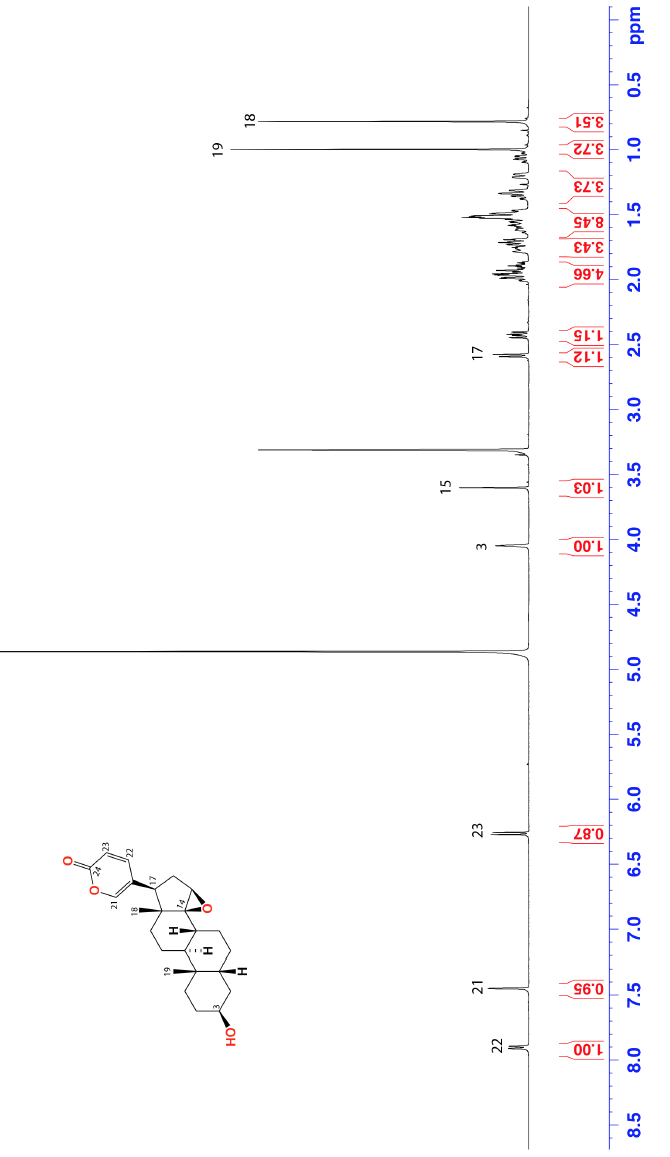


**Figure S4.** ^1^H NMR (CD_3_OD, 600 MHz) spectrum of resibufagin (**4**)


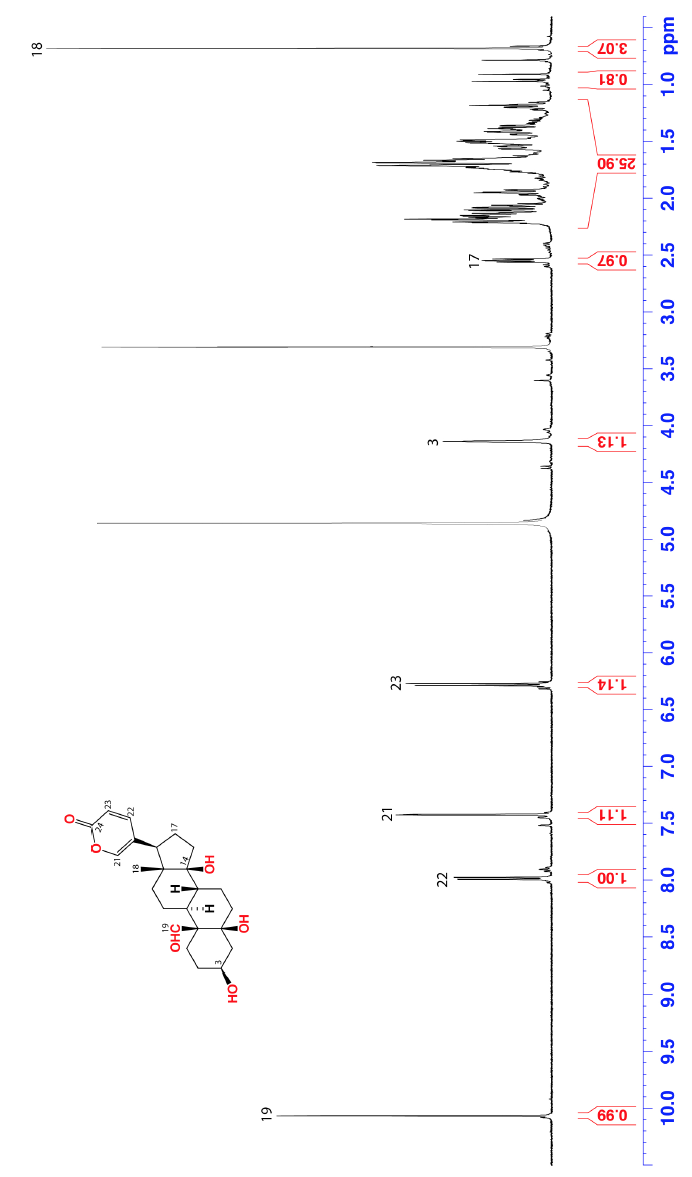


**Figure S5.** ^1^H NMR (CD_3_OD, 600 MHz) spectrum of hellebrigenin (**5**)


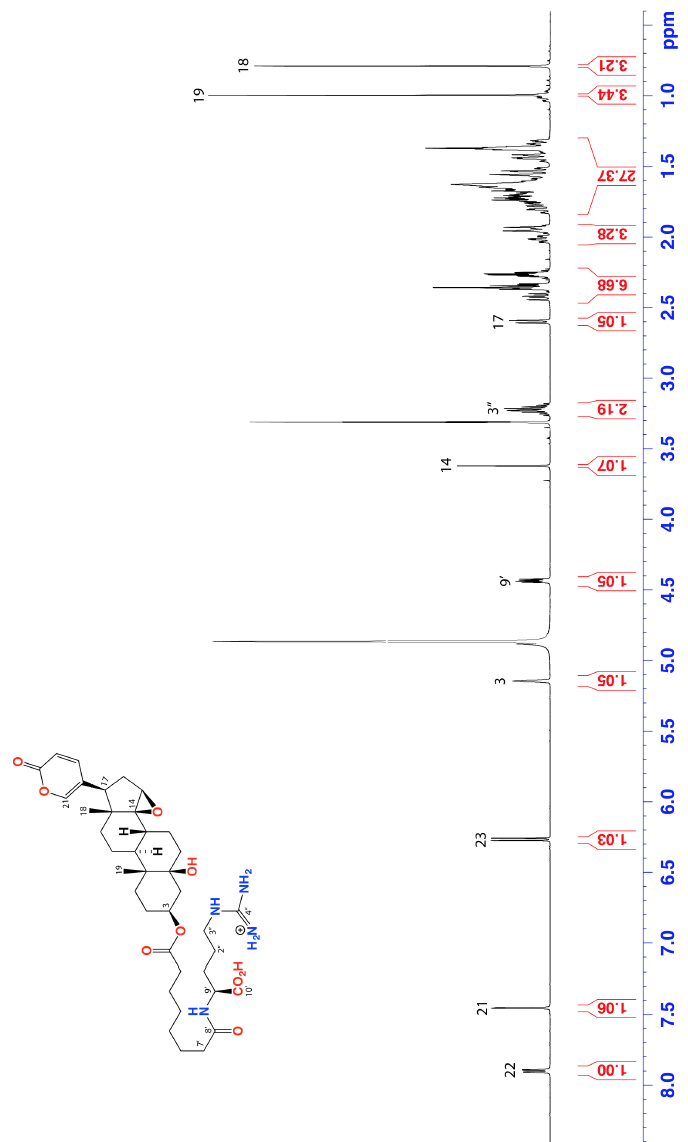


**Figure S6.** ^1^H NMR (CD_3_OD, 600 MHz) spectrum of marinobufotoxin (**6**)


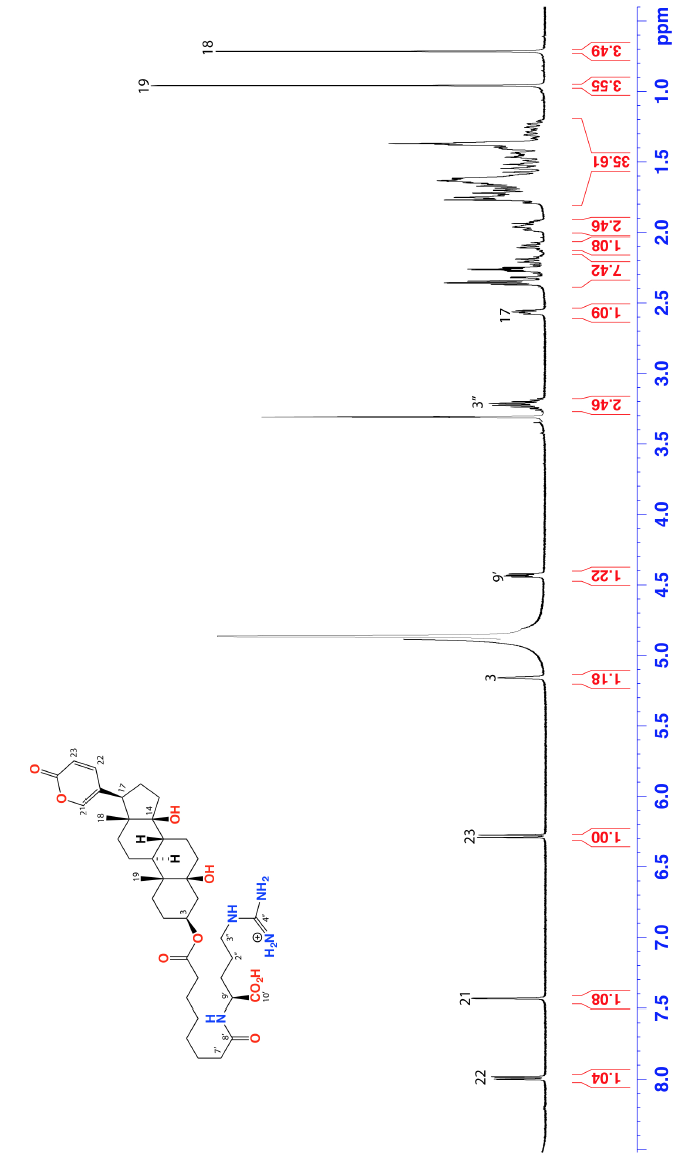


**Figure S7.** ^1^H NMR (CD_3_OD, 600 MHz) spectrum of telocinobufotoxin (**7**)


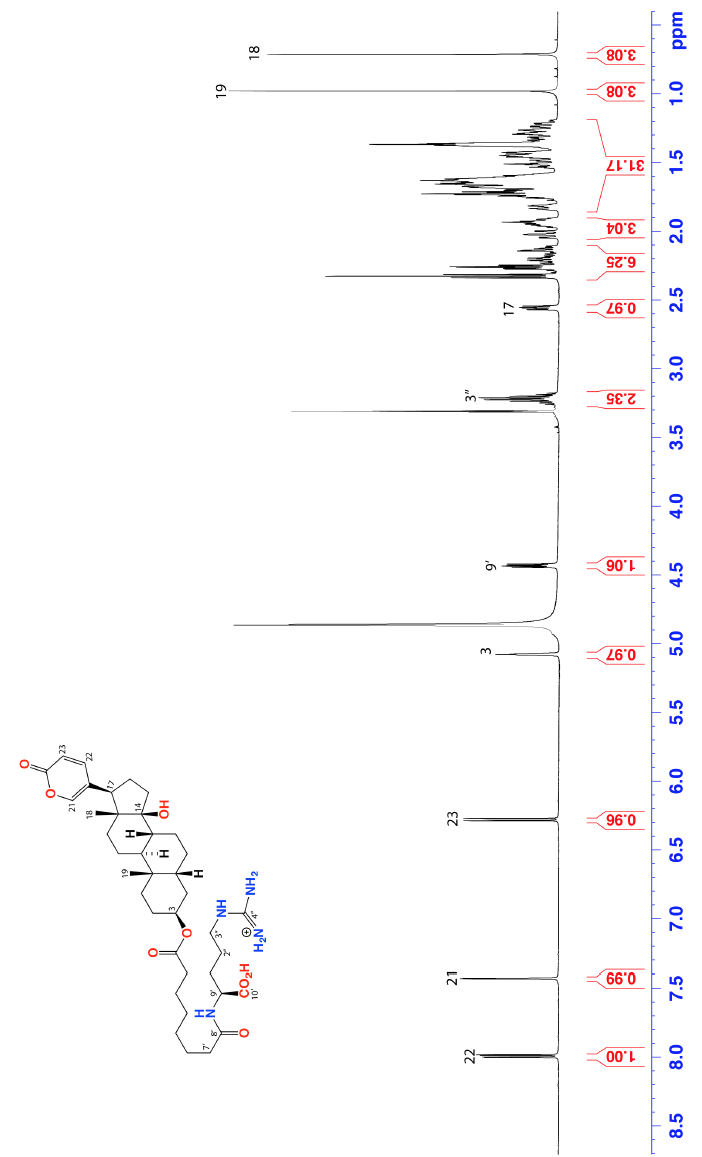


**Figure S8.** ^1^H NMR (CD_3_OD, 600 MHz) spectrum of bufalitoxin (**8**)


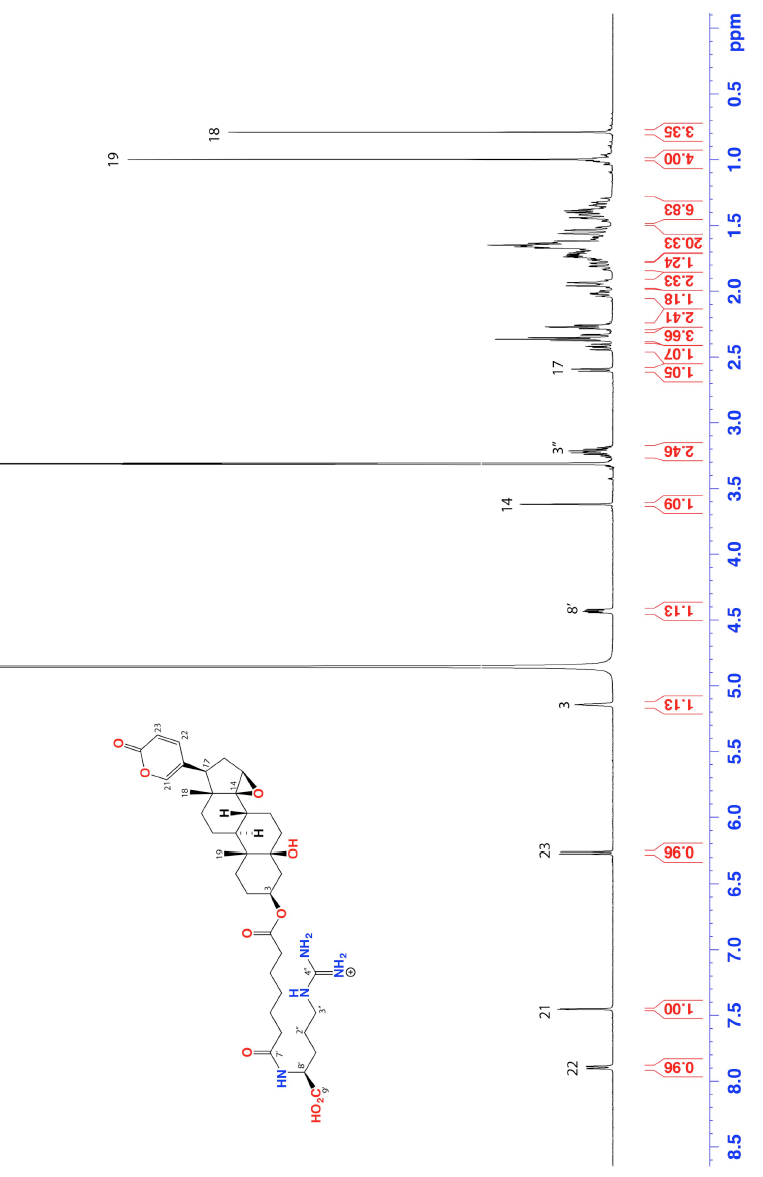


**Figure S9.** ^1^H NMR (CD_3_OD, 600 MHz) spectrum of marinobufagin-3-pimeloyl-L-arginine (**9**)

**Figure S10.** ^1^H NMR (CDCl_3_, 600 MHz) spectrum of bufolipin (**10**)


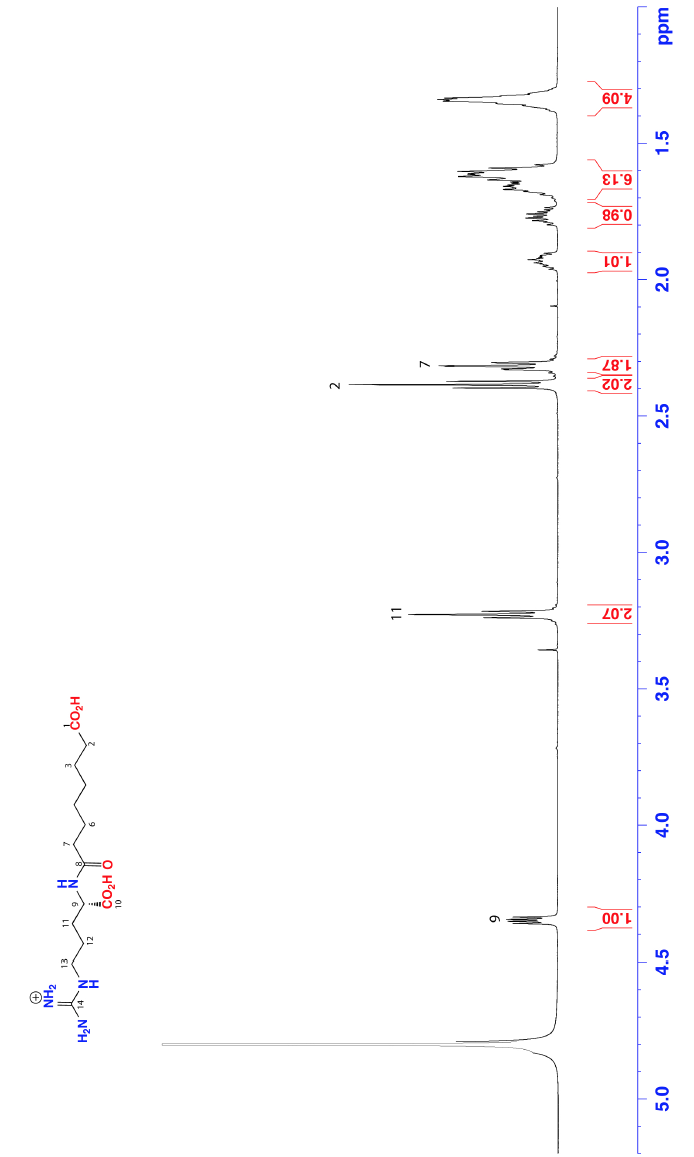


**Figure S11.** ^1^H NMR (CD_3_OD, 600 MHz) spectrum of suberoyl-L-arginine (**13**)

**Table S1. Identification of compounds observed in Figures 1a-1e**

| **#** | Compound name | LRMS^a^ | HRESIMS^b^ | Molecular formula |
| --- | --- | --- | --- | --- |
| **1** | Marinobufagin | 401.2 [M+H]^+^ | 401.2323 | C_24_H_32_O_5_ |
| **2** | Telocinobufagin | 403.2 [M+H]^+^ | 403.2479 | C_24_H_34_O_5_ |
| **3** | Bufalin | 387.3 [M+H]^+^ | 387.2530 | C_24_H_34_O_4_ |
| **4** | Resibufagin | 385.5 [M+H]^+^ | 385.2330 | C_24_H_32_O_4_ |
| **5** | Hellebrigenin | 417.2 [M+H]^+^ | 417.2272 | C_24_H_32_O_6_ |
| **6** | Marinobufotoxin | 713.4 [M+H]^+^ | 713.4129 | C_38_H_56_N_4_O_9_ |
| **7** | Telocinobufotoxin | 715.4 [M+H]^+^ | 715.4327 | C_38_H_58_N_4_O_9_ |
| **8** | Bufalitoxin | 699.4 [M+H]^+^ | 699.4327 | C_37_H_58_N_4_O_8_ |
| **9** | Hellebritoxin | 729.4 [M+H]^+^ | 729.4071 | C_38_H_56_N_4_O_10_ |
| **10** | Marinobufagin-3-pimeloyl-L-arginine | 699.4 [M+H]^+^ | 699.4108 | C_37_H_54_N_4_O_9_ |
| **11** | Bufolipin A | 657.4 [M-H]^-^ | 657.4009 | C_38_H_58_O_9_ |
| **12** | Dehydrobufotenin | 203.1 [M+H]^+^ |  | C_12_H_14_N_2_O |

^a^Mass observed in Agilent LCMS. ^b^Mass obtained from Bruker HRMS for authentic sample

**Supplementary Figures**


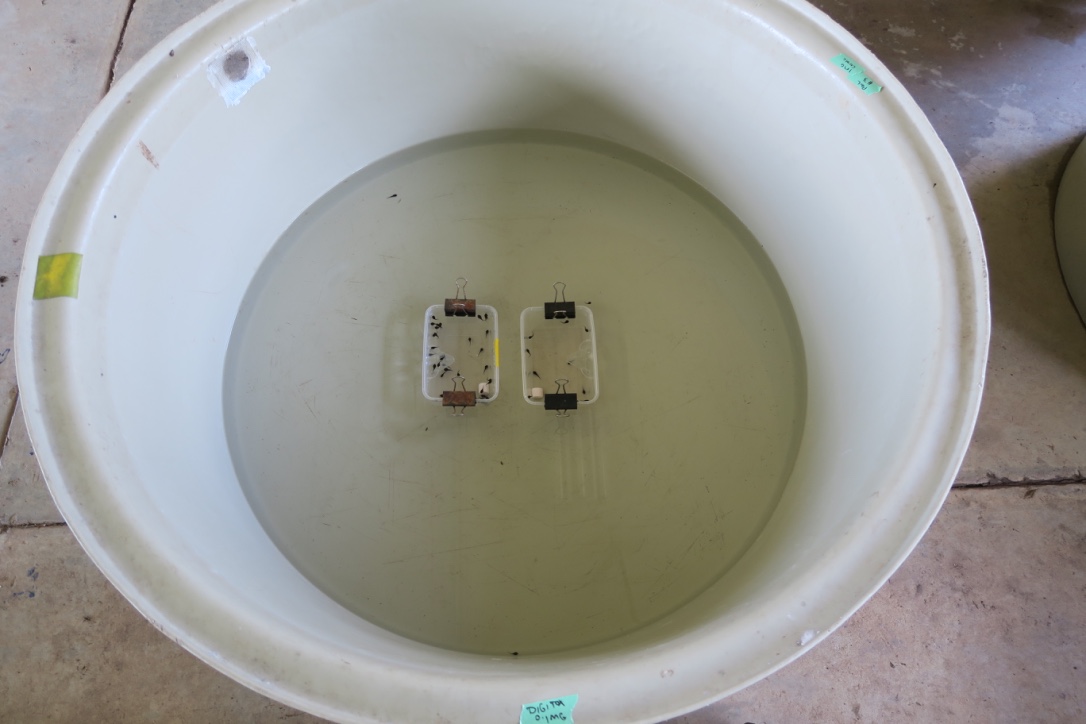


**Supplementary Figure 1.** Experimental pool set-up to test attraction response of toad tadpoles to chemical cues. Each pool was stocked with 50 tadpoles. Photo is of attraction response to toad egg extract (0.1 mg). The trap on the left contains the chemical bait, the trap on the right contains the control bait.


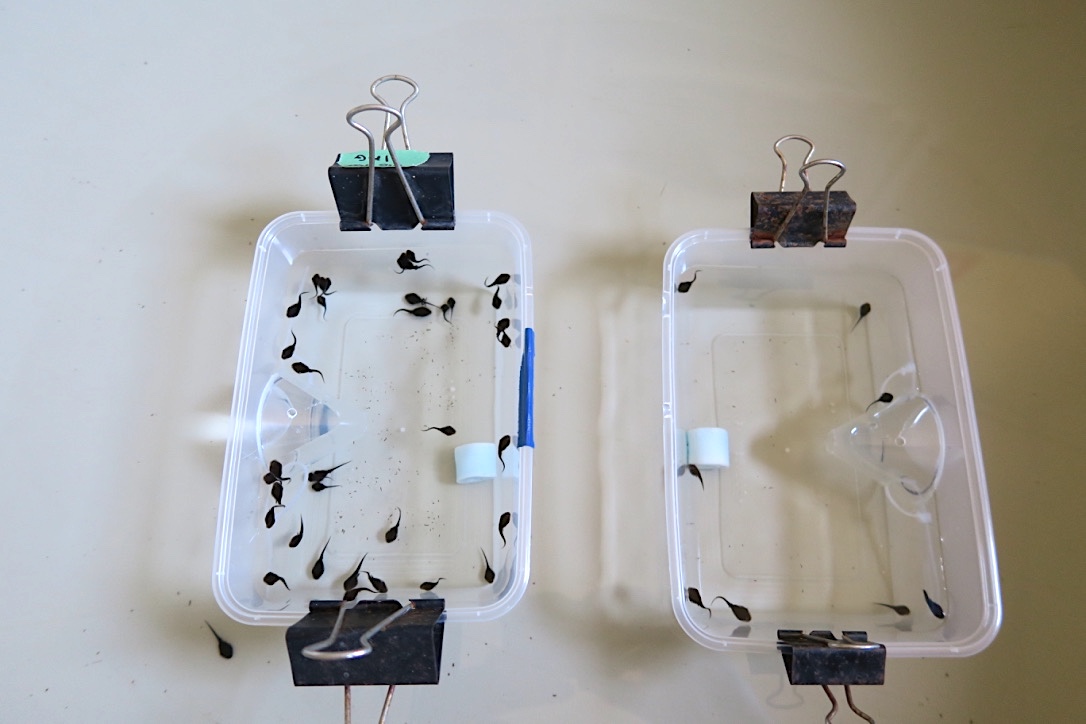


**Supplementary Figure 2.** Attraction response of toad tadpoles to marinobufagin (2.5 mmoles). The trap on the left contains the chemical bait, the trap on the right contains the control bait.


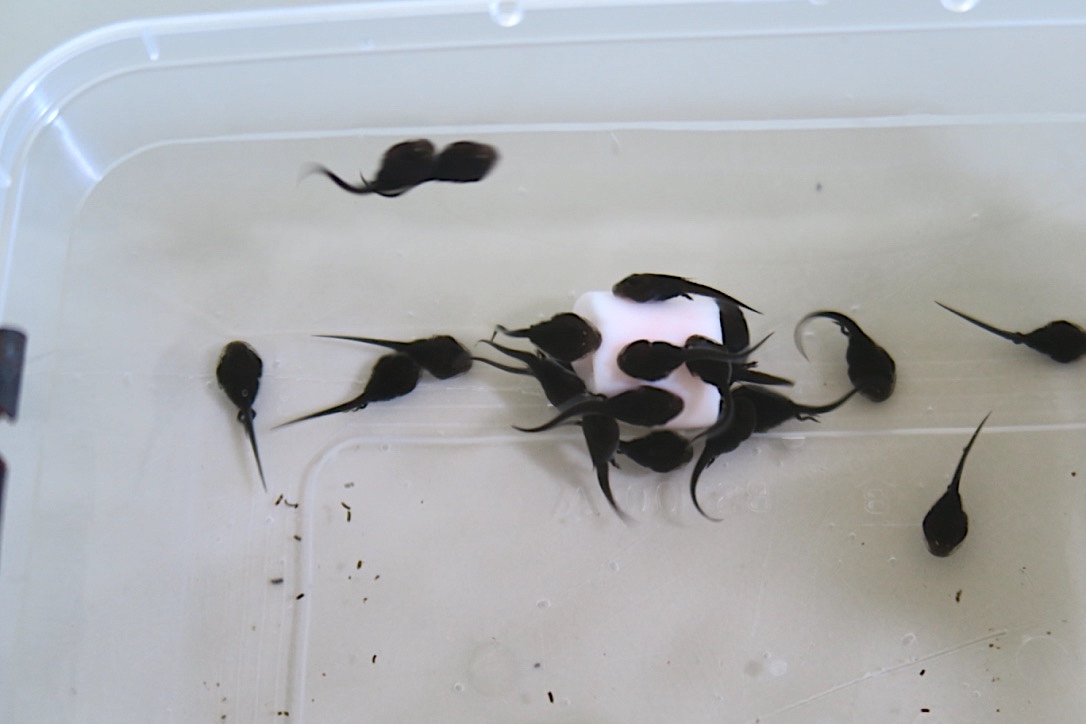


**Supplementary Figure 3.** Attraction response of toad tadpoles to egg extract (0.1 mg) bait. Tadpoles were actively feeding on the bait. In contrast, no such feeding response was observed towards control baits in any of the trials.
